# The association between the triglyceride-glucose index and sarcopenia: data from the NHANES 2011–2018

**DOI:** 10.1186/s12944-024-02201-1

**Published:** 2024-07-19

**Authors:** Jiju Yang, Cong Liu, Sihao Zhao, Lixiang Wang, Guanwei Wu, Ziyi Zhao, Chungen Li

**Affiliations:** 1 Beijing University of Chinese Medicine, Beijing, China; 2 Beijing Hospital of Traditional Chinese Medicine, Capital Medical University, Beijing, China

**Keywords:** Triglyceride-glucose index, Sarcopenia, Insulin resistance, NHANES, Cross-sectional study

## Abstract

**Background:**

The Triglyceride-glucose (TyG) index is a marker of insulin resistance, but its role in sarcopenia is controversial. The purpose of this study was to investigate the association of the TyG index with sarcopenia.

**Methods:**

4030 participants aged 20 years and above were selected from National Health and Nutrition Examination Survey for cross sectional study. Weighted logistic regression model was used to estimate the association between TyG index and sarcopenia. Threshold effect analysis and restricted cubic spline were employed to describe nonlinear link, with interaction tests and subgroup analyses performed.

**Results:**

It was found in the fully adjusted model that the TyG index was positively associated with sarcopenia (per 1-unit increase in the TyG index: OR = 1.31, 95%CI: 1.07, 1.60). This association was further highlighted in groups characterized by the absence of MetS or diabetes, as well as the absence of vigorous or moderate work activity. Furthermore, analysis of the curve fitting and threshold effects indicated a nonlinear relationship, which exhibited a turning point at 9.14.

**Conclusion:**

The study results indicated that the TyG index was positively associated with sarcopenia. Enhancing the management of insulin resistance could help reduce the risk of developing sarcopenia.

**Supplementary Information:**

The online version contains supplementary material available at 10.1186/s12944-024-02201-1.

## Introduction

Sarcopenia is a progressive disease that is characterized by a reduction in the quantity and quality of skeletal muscle [1]. It is especially common among older individuals, with reported incidence rates varying widely from 5 to 50% [2, 3]. Global population ageing is leading to a rise in the incidence of sarcopenia. [4]. Sarcopenia increases the risk of fractures and falls and may even lead to death [5]. Additionally, studies have shown that the direct medical expenses attributed to sarcopenia amount to as much as $18.5 billion [6]. Sarcopenia clearly represents a significant public health threat and economic burden to health care systems. Therefore, the early identification and prevention of sarcopenia are of paramount importance. Currently, the diagnosis of sarcopenia relies on methods such as CT or dual-energy X-ray absorptiometry (DXA), along with measurements of grip strength or walking speed as physiological parameters [7]. However, in a busy clinical setting, these examinations can be expensive, nonportable, and complex to perform. It is of the utmost importance to identify a biomarker that is straightforward, reliable, precise, and economical and can be used for the early detection and prediction of sarcopenia in individuals at increased risk.

As investigations into the causes of sarcopenia continue, insulin resistance (IR) has become a focal point in relation to this disease [8]. Many studies have indicated that IR is a pivotal element in the degradation of skeletal muscle tissue and may be related to factors such as the accumulation of adipocytes [9], chronic inflammation [10], and the activation of AMP-activated protein kinase [11]. The triglyceride-glucose (TyG) index, which is widely used as a reliable measure of IR and is derived from measurements of fasting glucose and triglyceride levels [12], is characterized by its straightforwardness and economic efficiency. It has shown high diagnostic and predictive value in diseases such as arthritis [13], kidney stones [14], and depression [15]. Epidemiological studies that are currently underway have produced varied results regarding the link between sarcopenia and the TyG index. A study revealed a notable positive correlation between the TyG index and muscle mass in Korean individuals [16], whereas another cohort investigation revealed a negative correlation in elderly individuals from China [17]. This study is the first to use National Health and Nutrition Examination Survey (NHANES) data to investigate the association of the TyG index with sarcopenia among Americans, with the aim of contributing new insights to the field of sarcopenia prevention.

## Methods

### Study population

The data were obtained from the NHANES. The NHANES collects comprehensive data on nutritional intake, health status, lifestyle factors, and other aspects in adult and paediatric populations in the United States. It employs complex sampling methods to ensure the representativeness of the surveyed population. Informed consent was obtained from all participants in the NHANES, which was ethically approved.

**Fig. 1:**
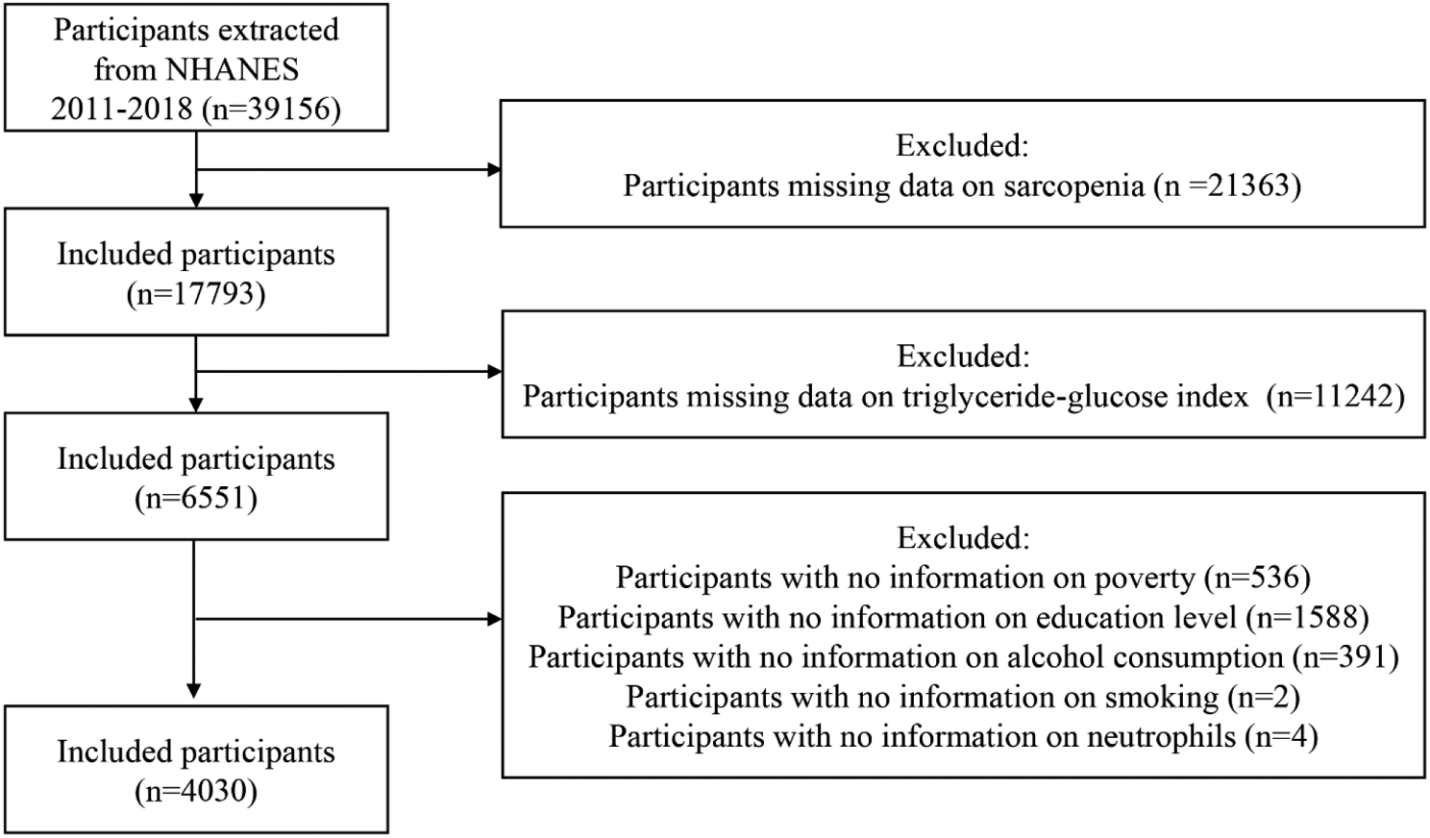
Flowchart for inclusion of study participants

Data from the NHANES spanning eight consecutive years were included, covering the 2011–2012, 2013–2014, 2015–2016, and 2017–2018 cycles, with a total of 39,156 participants. We first excluded participants missing DXA data and body mass index (BMI) data due to the inability to determine whether these individuals had sarcopenia. Next, participants lacking triglyceride and glucose data were excluded because the absence of these data precluded the calculation of the TyG index. Subsequently, participants missing family poverty income ratio (PIR) data (536), educational information (1,588), alcohol consumption information (391), smoking information (2) and information on neutrophils (4) were also excluded, leaving 4,030 participants for inclusion. The detailed screening process is shown in Fig. 1.

### Definition of the TyG index and sarcopenia

The TyG index was determined by the following formula: TyG = ln [fasting triglycerides (mg/dL) × fasting glucose (mg/dL)/2] [18].

DXA was employed to measure the appendicular skeletal muscle mass (ASM), which represents the aggregate lean mass in the arms and legs, but it was unable to be used for individuals who were pregnant, who were taller than 192.5 cm, or who were weighed more than 136.4 kg.

The sarcopenia index is defined as the ASM divided by BMI. A sarcopenia index of < 0.512 for females and < 0.789 for males indicates sarcopenia [19].

### Assessment of covariates

The demographic data included sex, age, race, education level, PIR and marital status. BMI could be found in the examination data. The numbers of neutrophils and lymphocytes could be found in the laboratory data. The neutrophil-to-lymphocyte ratio (NLR) was determined by the following formula: NLR = neutrophil count/lymphocyte count. Diabetes duration is also closely related to IR, but this covariate could only be calculated in diabetic; for non-diabetic, the diabetes duration was set to zero. In this study, 88% of participants had a duration of zero, leading to a significant skew in the data distribution. To ensure the stability of the results, this covariate was not ultimately included. Comprehensive definitions of smoking status, alcohol consumption status, hypertension status, diabetes status, metabolic syndrome status (MetS), vigorous work activity status, moderate work activity status and use of glucocorticoids status are provided in Additional file: Table S1.

### Statistical analysis

R software (R version 4.3.1) was used for the statistical analysis. *P* < 0.05 was considered to indicate statistical significance. The NHANES employs a sophisticated sampling methodology, which is complemented by sample weights, to ensure that the data collected are reflective of the national population. The data were adjusted using the sample weight calculation approach suggested by the NHANES, with the following specific calculation method: Weights = WTMEC2YR/4. In the weighted baseline table, for continuous data, the mean ± standard deviations are provided, and for categorical data, percentages are given. Continuous variables were tested using the Kruskal‒Wallis H test, while categorical variables were analysed with chi-squared tests. The associations between the TyG index and sarcopenia were investigated through weighted multivariate logistic regression models, and the results are shown as odds ratios (ORs) with 95% confidence intervals (95% CIs). Model 1 did not involve any adjustments for covariates. Model 2 controlled for sex, age, race, marital status, education level, and PIR. Model 3 included all the covariates in the analysis. According to the multivariate logistic regression analysis, the TyG index served as both a continuous variable and a categorical variable. The study included subgroup analyses that explored the impact of sex, age, BMI, diabetes status, MetS status, vigorous work activity status, and moderate work activity status. An exploration of the relationship of the TyG index with sarcopenia was carried out using weighted restricted cubic spline (RCS), including an assessment of the threshold effect. Additionally, to validate the stability of the results, after employing multiple imputation with 5 replications and the chained equation to handle missing data, the RCS results were compared between the imputed dataset and the dataset with original missing values. To assess multicollinearity, the variance inflation factor (VIF) was computed. A VIF < 10 indicated that there was no multicollinearity among the covariates.

## Results

### Participants’ baseline characteristics

The sample for the study was composed of 4030 individuals, including 2012 males and 2018 females. Among the included individual, 336 had sarcopenia, for an incidence rate of 8.34%. Compared to individuals without sarcopenia, those with sarcopenia tended to be older, Mexican-American, hypertensive, diabetic, obese, on glucocorticoids and have MetS. They had relatively lower education and income levels but higher triglyceride levels, glucose levels, TyG index values, neutrophil counts and lymphocyte counts. The baseline characteristics are shown in Table 1.

**Table 1:** Weighted baseline characteristics by Sarcopenia

Characteristic	Non-Sarcopenia (*n* = 3694)	Sarcopenia (*n* = 336)	*P*-value
Sex (%)			0.248
Male	50.68	55.40	
Female	49.32	44.60	
Age (years)	39.07 (11.77)	42.96 (10.96)	< 0.001
Race (%)			< 0.001
Mexican American	9.20	24.09	
Non-Hispanic White	64.79	52.92	
Non-Hispanic Black	10.78	3.19	
Other	15.22	19.80	
Marital status (%)			0.796
Married/Living with partner	62.05	62.95	
Widowed/Divorced/Separated/ Never married	37.95	37.05	
Education level (%)			< 0.001
Less than high school	11.67	23.25	
High school diploma	21.33	24.56	
More than high school	67.00	52.19	
Poverty Income Ratio (%)			< 0.001
< 1.3	22.75	36.35	
1.3–3.5	35.54	36.19	
>3.5	41.70	27.46	
Hypertension status (%)			< 0.001
Yes	26.69	43.89	
No	73.31	56.11	
Diabetes status (%)			< 0.001
Yes	9.46	23.52	
No	90.54	76.48	
Metabolic syndrome status (%)			< 0.001
Yes	26.04	52.62	
No	73.96	47.38	
Smoking status (%)			0.328
Now	22.11	19.09	
Former	20.16	24.24	
Never	57.73	56.67	
Alcohol consumption status (%)			< 0.001
Now	83.74	66.04	
Former	7.83	16.28	
Never	8.43	17.68	
Body mass index (%)			< 0.001
Normal	33.72	6.10	
Overweight	34.16	23.16	
Obesity	32.13	70.74	
Vigorous work activity status (%)			0.368
Yes	25.30	22.70	
No	74.70	77.30	
Moderate work activity status (%)			0.008
Yes	45.55	33.27	
No	54.45	66.73	
Neutrophils (1000 cells/µL)	3.92 ± 1.53	4.66 ± 1.94	< 0.001
Lymphocyte (1000 cells/µL)	2.07 ± 0.65	2.25 ± 0.67	0.001
Neutrophil-to-lymphocyte ratio	2.02 ± 0.93	2.21 ± 1.04	0.031
Use of glucocorticoids status (%)			0.006
Yes	1.17	4.61	
No	98.83	95.39	
Triglyceride (mg/dl)	116.97 ± 98.05	152. 34 ± 105.72	< 0.001
Glucose (mg/dl)	103.55 ± 29.07	115.71 ± 42.81	0.001
Triglyceride-glucose index	8.48 ± 0.69	8.88 ± 0.67	< 0.001

### Associations between the TyG index and sarcopenia

Before the analyses, the study evaluated the collinearity. The VIF for all covariates was less than 10, indicating that there was no multicollinearity. (Additional file: Table S2)

Three different model analyses revealed that regardless of a one-unit increase or a one-SD increase, the TyG index was positively associated with the likelihood of developing sarcopenia. In model 3, after converting the TyG index into a categorical variable by quartiles, the results showed that, compared to that in the lowest quartile group, the risk of sarcopenia in the top quartile group was 2.46 times greater (OR = 2.46, 95%CI: 1.36, 4.45). (Table 2)

**Table 2:** Associations of the TyG index with the risk of Sarcopenia

TyG index	OR (95% CI)		
	Model 1	Model 2	Model 3
Per 1 unit increase	2.08 (1.78, 2.41) ^**^	1.73 (1.47, 2.05) ^**^	1.31 (1.07, 1.60) ^*^
Per 1 SD increase	1.68 (1.51, 1.87) ^**^	1.48 (1.31, 1.66) ^**^	1.21 (1.05, 1.40) ^*^
Q1	Ref	Ref	Ref
Q2	2.27 (1.31, 3.94) ^**^	1.91 (1.12, 3.26) ^*^	1.58 (0.86, 2.90)
Q3	4.41 (2.61, 7.48) ^**^	3.34 (1.95, 5.71) ^**^	2.18 (1.19, 4.02) ^*^
Q4	6.58 (3.95, 10.96) ^**^	4.39 (2.58, 7.45) ^**^	2.46 (1.36, 4.45) ^*^
*P*-trend	< 0.001	< 0.001	0.002

Model 1: No covariates were adjusted; Model 2: Adjusted for sex, age, race, education level, PIR, and marital status; Model 3: Adjusted for all variables: sex, age, race, education level, PIR, marital status, BMI, smoking status, alcohol consumption status, hypertension status, diabetes status, metabolic syndrome status, vigorous work activity status, moderate work activity status, NLR and Use of glucocorticoids status

* *P* < 0.05

** *P* < 0.001

Model 1 sample size: 4030; Model 2 sample size: 4030; Model 3 sample size: 4025

### Subgroup analyses

**Fig. 2:**
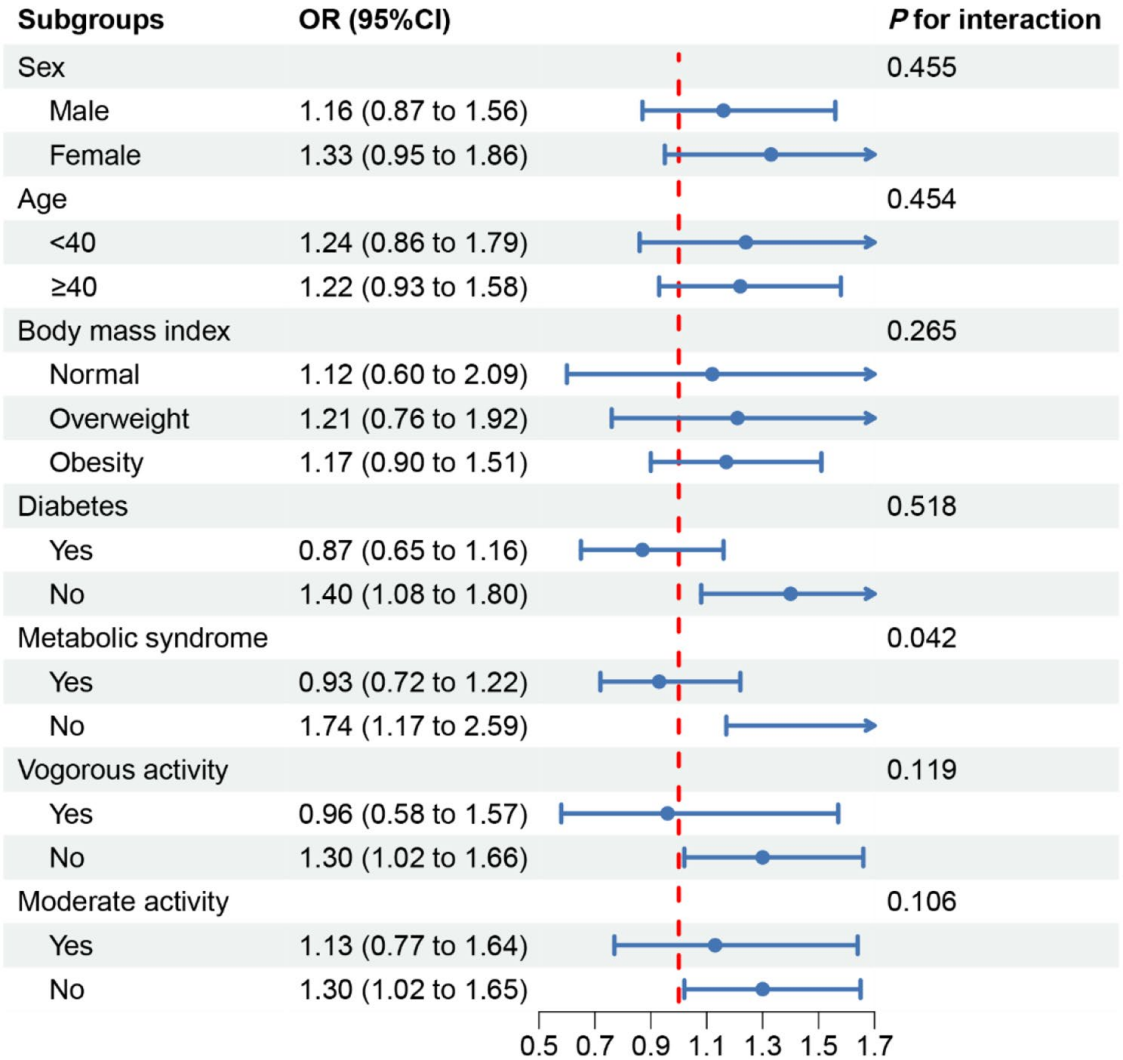
Subgroup analysis and interaction analysis

Subgroup analyses were conducted based on sex, age, BMI, diabetes status, MetS status, vigorous work activity status, and moderate work activity status. The TyG index exhibited significant positive correlations with sarcopenia only in the subgroups of participants who did not have MetS or diabetes and did not perform vigorous or moderate work-related activities (*P* < 0.05). The results of the subgroup analyses are shown in Fig. 2.

### Nonlinear association between the TyG index and sarcopenia

**Fig. 3:**
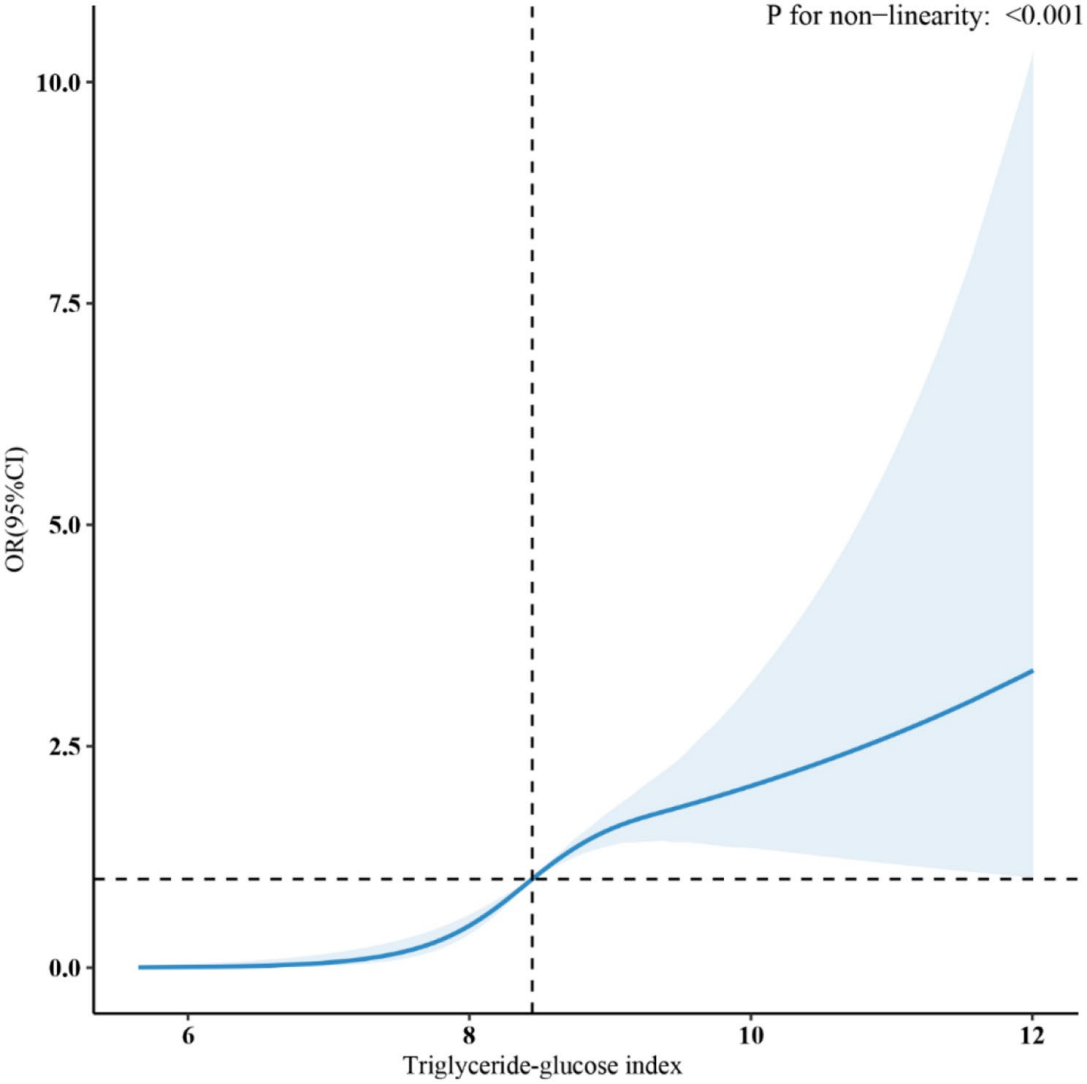
Analysis of restricted cubic spline sarcopenia

The relationship between the TyG index and sarcopenia, as determined by RCS, was nonlinear (Fig. 3). The segmented regression model was employed to calculate the threshold effect across each interval. The inflection point of the TyG index was 9.14. When the TyG index was below 9.14, there was no significant association (OR = 1.30, 95%CI: 0.80, 2.10); when the TyG index exceeded 9.14, a positive association was observed (OR = 3.56, 95%CI: 2.62, 4.83) (Table 3).

**Table 3:** Threshold effect analysis

Outcomes	Breakpoint.OR (95%Cl)	*P* value
Inflection point (K)	9.14 (9.11, 9.18)	
OR1, (95% CI) (> K), *p* value	3.56 (2.62, 4.83)	< 0.001
OR1, (95% CI) (< K), *p* value	1.30 (0.80, 2.10)	0.291
Likelihood Ratio test	-	< 0.001

### Sensitivity analysis

**Fig. 4:**
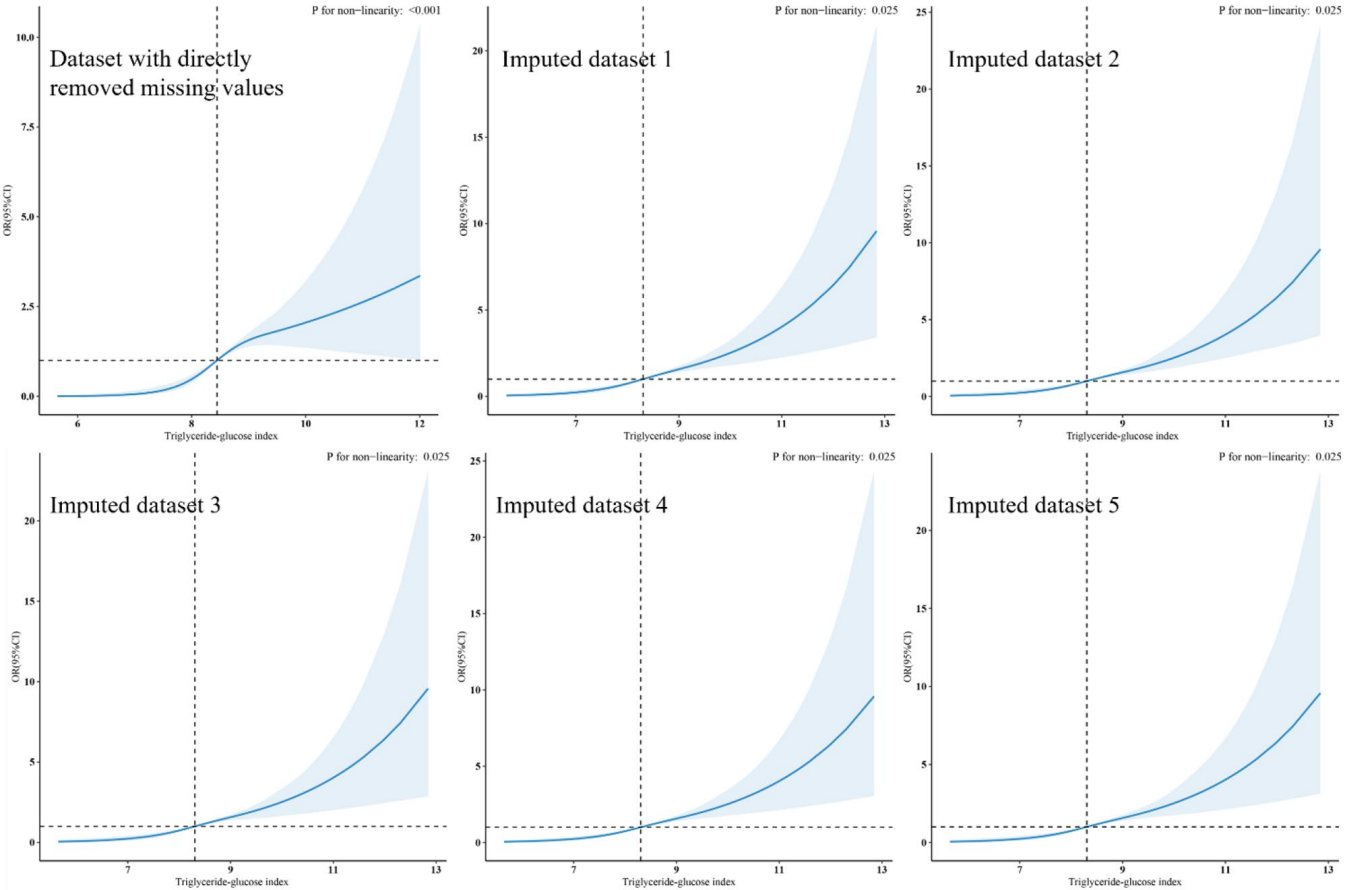
Sensitivity analysis

Five different datasets were obtained by imputing missing covariates. When these five interpolation datasets were assessed using RCSs, their outcomes were observed to align consistently with the results obtained from the dataset after removing missing values. (Fig. 4)

## Discussion

The present study investigated the association between the TyG index and sarcopenia by employing an NHANES dataset and including 4,030 individuals. Weighted multivariable logistic regression revealed a notable positive association between the TyG index and the presence of sarcopenia. This association remained robust after adjusting for all covariates, including sex, age, race, and others. Furthermore, the TyG index was found to have a more intense relationship with sarcopenia in the subgroups characterized by the absence of MetS or diabetes, as well as the absence of vigorous or moderate work activity. RCS analysis also supported the nonlinear association between the TyG index and sarcopenia. The results suggested that regular monitoring of the TyG index may help in the early prevention of sarcopenia.

The precise mechanisms by which the TyG index correlates with sarcopenia are not yet completely understood. However, IR is considered to play a significant role in this process. IR refers to a pathological state in which insulin absorption and glucose utilization decrease, resulting in the failure to meet the needs of the body’s target tissues and leading to a compensatory increase in insulin secretion [20]. Existing research has confirmed a close association between IR and sarcopenia, with both potentially causing each other and forming a vicious cycle. IR can promote the development of sarcopenia by increasing the degradation of muscle proteins [21], reducing protein synthesis, upregulating the expression of the FoxO family [22, 23], and activating autophagy in skeletal muscle cells [24]. Conversely, sarcopenia may further exacerbate IR through abnormal fat distribution and infiltration [25], increased production of branched-chain amino acid metabolites [26, 27], and a decreased proportion of skeletal muscle type I fibres [28]. In addition, mitochondrial dysfunction, endoplasmic reticulum stress, and the activation of inflammatory factors are also considered important factors that exacerbate the development of both sarcopenia and IR [29].

Clinically, hyperinsulinaemic-euglycemic clamps (HECs) and homeostasis model assessment of IR (HOMA-IR) are commonly employed to evaluate an individual’s level of IR. However, the invasive nature and high costs of HECs limit their widespread clinical application [30], and HOMA-IR requires the measurement of fasting insulin levels, which may prevent some smaller medical facilities from conducting the test, affecting the broad application of HOMA-IR [31]. To address these issues, the TyG index has been considered a credible barometer in recent years [32]. Insulin enhances the removal of glucose from the bloodstream and its utilization by the liver. However, IR leads to elevated levels of plasma glucose and promotes the conversion of excessive glucose into triglycerides in the liver. Furthermore, IR increases the generation of free fatty acids, which are then transformed into hepatic triglycerides [33, 34]. The TyG index not only has high accuracy in predicting arterial stiffness in patients with diabetes and metabolic syndrome [35–37] but also, due to the shared risk factors between IR and sarcopenia, such as metabolic syndrome and diabetes, has the potential to serve as a tool for assessing the risk of sarcopenia associated with IR.

The relationship between the TyG index and sarcopenia is controversial. The results from two Korean studies derived from the Korea NHANES suggest that as the TyG index increases, muscle mass tends to decrease [16, 38]. However, a study conducted in China showed that as the TyG index increased, the likelihood of developing sarcopenia decreased, although this association did not become more pronounced after accounting for BMI [17]. This inconsistency could be explained by variations in sample size, ethnicity, and research methodology and the discrepancies in databases employed across these investigations. Furthermore, the aforementioned three studies were conducted among individuals aged 40 years and older, nondiabetic individuals, and individuals aged 60 years and older, thus limiting the generalizability of the results. Studies have shown that among every 10 young people, at least one person has sarcopenia [39], and diabetic patients are also at high risk for sarcopenia [40]. Second, these studies had limitations in terms of controlling for potential confounding factors. For example, important factors such as physical activity levels and the use of glucocorticoid medications were not adequately considered, which could affect the relationship between muscle mass and IR. In particular, changes in the level of physical activity might significantly influence muscle mass and metabolic health but were not thoroughly evaluated in these studies. Although these studies provided important insights into the relationship between the TyG index and sarcopenia, their findings might be limited by the characteristics of the specific populations studied, which restricts the general applicability of the results. This study involved a broader age range and included participants with diabetes, considering important covariates such as BMI, physical activity levels, glucocorticoid use, and infection status, which maximizes the reduction in the influence of confounding factors and, compared to previous studies, is more representative and accurate. Furthermore, a threshold effect analysis was also conducted in this study, providing a reference for the development of strategies to prevent sarcopenia in the future.

In the subgroups of participants who did not have MetS or diabetes and perform vigorous or moderate work activities was there a substantial positive correlation between the TyG index and sarcopenia. MetS is a multifactorial metabolic disorder encompassing obesity, hyperglycemia, hypertension, and hyperlipidemia [41]. As a result of these factors, individuals suffering from MetS typically exhibit elevated levels of blood glucose and lipids. Within this context, there was no significant correlation between the TyG index and sarcopenia among MetS patients. But, in the non- MetS population, a higher TyG index raised sarcopenia risk. Furthermore, diabetes caused by metabolic dysfunction is characterized by IR [42], which impairs the response of diabetes patients to insulin and consequently attenuates the correlation between the TyG index and sarcopenia, rendering this relationship less pronounced in diabetes individuals compared to non-diabetic individuals. However, the role of the TyG index in patients without MetS or diabetes suggests that metabolic factors potentially occupy a pivotal position in the development of sarcopenia. Past studies have shown that the incidence of sarcopenia was relatively high among patients with Mets [43, 44], and the findings of this study further validated this viewpoint. Apart from Mets, liver lipid accumulation triggering non-alcoholic fatty liver disease (NAFLD) also increments the risk of sarcopenia [45]. Notably, the TyG index positively correlates with NAFLD risk [46], likely due to its components—serum triglycerides and glucose—being metabolic factors in fatty liver development [47]. Therefore, there are also reasons to believe that metabolic health may affect other muscle-skeletal degenerative diseases [48, 49]. Given the differential effects of the TyG index across different metabolic states, this suggests that individual metabolic status should be considered in sarcopenia pathogenesis research. It is well known that physical activity can increase muscle mass and improve muscle function; furthermore, exercise helps to improve IR [50]. In the subgroup of participants who did not engage in vigorous or moderate work activity, a lack of physical activity may have led to a decline in muscle mass and an exacerbation of IR, which could be one of the reasons for the stronger observed correlation. This discovery provided important scientific evidence for the development of exercise-specific prevention strategies for sarcopenia. However, due to the smaller sample size in specific subgroups, caution should be considered regarding the results.

## Strengths and limitations

This study had the following advantages. One of the key strengths of this study was that it is the first to explore the correlation between the TyG index and sarcopenia within the American population based on NHANES data. The NHANES employs a probability stratified sampling method, allowing the data to adequately reflect health status and nutritional levels across individuals with different socioeconomic backgrounds in the United States. Second, to minimize the influence of confounding factors, this study adjusted for possible covariates. Furthermore, subgroup analyses were conducted to examine how other factors might influence the relationship between the TyG index and sarcopenia. Sensitivity analyses were carried out using five different datasets derived from imputation to ensure the stability of the results.

This study also had a few limitations. First, the cross-sectional design of the study restricted its capacity to establish a confirmed causal relationship between the TyG index and sarcopenia. Second, although this study adjusted for many covariates, other factors, such as medication use, the severity of related diseases, or insulin dependence, were not included. Therefore, the possibility of undetected confounding variables affecting the outcomes cannot be ruled out. Besides, some of the covariates were also collected with the risk of recall bias. Moreover, although the present study was conducted among an American cohort, triglyceride levels exhibit variation across different nations and ethnicities [51], which may restrict the applicability of the results to diverse populations.

## Conclusion

In summary, the results indicated a significant positive correlation between the TyG index and the incidence of sarcopenia in the US population. This finding validated the use of the TyG index, suggesting its utility as a prospective indicator for predicting the development of sarcopenia. This study highlighted the importance of IR in the pathogenesis of sarcopenia. By regularly monitoring the TyG index, clinicians might be able to identify individuals at high risk for sarcopenia early, allowing for the implementation of targeted interventions. However, the specific molecular mechanisms underlying this relationship are not yet clear. Future research should focus on elucidating the potential biological pathways linking the TyG index and sarcopenia, as well as assessing whether reducing the TyG index through interventions could effectively reduce the incidence of sarcopenia.

### Electronic supplementary material

Below is the link to the electronic supplementary material.

Supplementary Material 1

## Data Availability

All data for this study can be found on the NHANES website.

## Abbreviations

BMI: Body mass index
CI: Confidence intervals
DXA: Dual-energy X-ray absorptiometry
HOMA-IR: Homeostasis model assessment of IR
HECs: Hyperinsulinaemic-euglycemic clamps
IR: Insulin resistance
MetS: Metabolic syndrome
NHANES: National Health and Nutrition Examination Survey
NAFLD: Non-alcoholic fatty liver disease
OR: Odds ratios
PIR: Poverty income ratio
RCS: Restricted cubic spline
SD: Standard deviation
TyG: Triglyceride-glucose
VIF: Variance inflation factor
