# Supplementary material for: The association between the triglyceride-glucose index and sarcopenia: data from the NHANES 2011–2018
Source: Lipids Health Dis. 2024 Jul 19;23:219. doi: 10.1186/s12944-024-02201-1 (PMC11264742; doi:10.1186/s12944-024-02201-1)
Supplement: Supplementary file 1 — Supplementary Material 1 [file 12944_2024_2201_MOESM1_ESM.docx]

| Additional file: Table S1 Definition of covariates | |
| --- | --- |
| Covariates | Definition |
| Smoking status |  |
| Now | smoked moth than 100 cigarettes in life and smoke some days or every day |
| Former | smoked less than 100 cigarettes in life |
| Never | smoked more than 100 cigarettes in life and smoke not at all now |
| Alcohol consumption status |  |
| Now | ≥1 drinks per day for females, ≥2 drinks per day for males, or binge drinking ≥2 |
| Former | had >12 drinks in 1 year and did not drink last year, or did not drink last year but drank>12 drinks in lifetime |
| Never | had <12 drinks in lifetime |
| Hypertension status | average systolic blood pressure ≥ 140 mmHg, an average diastolic blood pressure ≥ 90 mmHg; use hypertension medications; self-reported doctor's diagnosis of hypertension |
| Diabetes status | self-reported a doctor-diagnosed diabetes; taking antidiabetic medications |
| Metabolic syndrome status | if the following three or more conditions are met: ①Fasting glucose≥5.6 mmol/L (100 mg/dL) or drug treatment for elevated blood glucose; ②<1.0 mmol/L (40 mg/dL) (men); <1.3 mmol/L (50mg/dL) (women) or drug treatment for low HDL cholesterol; ③≥1.7 mmol/L (150 mg/dL) or drug treatment for elevated triglycerides; ④Waist ≥102 cm (men) or ≥88 cm (women); ⑤≥130/85 mmHg or drug treatment for hypertension. |
| Vigorous work activity status | work involves vigorous-intensity activity that causes large increases in breathing or heart rate like carrying or lifting heavy loads, digging or construction work for at least 10 minutes continuously |
| Moderate work activity status | work involves moderate-intensity activity that causes small increases in breathing or heart rate such as brisk walking or carrying light loads for at least 10 minutes continuously |
| Use of glucocorticoids status | use prednisone, prednisolone, methylprednisolone, and budesonide |

| Additional file: Table S2 The collinearity assessment outcomes | | | |
| --- | --- | --- | --- |
| variables | GVIF | df | GVIF^(1/(2*df)) |
| Sex | 1.236 | 1 | 1.112 |
| Age | 1.292 | 1 | 1.137 |
| Race | 1.449 | 3 | 1.064 |
| Marital status | 1.066 | 1 | 1.032 |
| Education level | 1.411 | 2 | 1.09 |
| Poverty Income Ratio | 1.361 | 1 | 1.166 |
| Hypertension status | 1.281 | 1 | 1.132 |
| Diabetes status | 1.395 | 1 | 1.181 |
| Metabolic syndrome status | 1.859 | 1 | 1.363 |
| Smoking status | 1.297 | 2 | 1.067 |
| Alcohol consumption status | 1.241 | 2 | 1.055 |
| Body mass index | 1.388 | 1 | 1.178 |
| Vigorous work activity status | 1.336 | 1 | 1.156 |
| Moderate work activity status | 1.278 | 1 | 1.130 |
| Neutrophil-to-lymphocyte ratio | 1.048 | 1 | 1.024 |
| Use of glucocorticoids status | 1.025 | 1 | 1.012 |
